# The interplay between interpersonal dynamics, treatment barriers, and larger social forces: an exploratory study of drug-using couples in Hartford, CT

**DOI:** 10.1186/1747-597X-1-12

**Published:** 2006-05-03

**Authors:** Janie Simmons

**Affiliations:** 1 National Development and Research Institutes/Medical and Health Research Association of NYC, NY, USA

## Abstract

**Background:**

The drug treatment field tends to place emphasis on the individual rather than the individual in social context. While there are a growing number of studies indicating that drug-using intimate partners are likely to play an important role in determining treatment options, little attention has been given to the experience and complex treatment needs of illicit drug-using (heroin, cocaine, crack) couples.

**Methods:**

This exploratory study used in-depth interviews and ethnographic engagement to better understand the relationship between interpersonal dynamics and the treatment experience of ten relatively stable drug-using couples in Hartford, CT. Semi-structured and open-ended qualitative interviews were conducted with each couple and separately with each partner. Whenever possible, the day-to-day realities and contexts of risk were also observed via participant and non-participant observation of these couples in the community. A grounded theory approach was used to inductively code and analyze nearly 40 transcripts of 60–90 minute interviews as well as fieldnotes.

**Results:**

This study builds on a concept of complex interpersonal dynamics among drug users. Interpersonal dynamics of care and collusion were identified: couples cared for each other and colluded to acquire and use drugs. Care and collusion operate at the micro level of the risk environment. Treatment barriers and inadequacies were identified as part of the risk environment at the meso or intermediate level of analysis, and larger social forces such as gender dynamics, poverty and the "War on Drugs" were identified at the macro level. Interpersonal dynamics posed problems for couples when one or both partners were interested in accessing treatment. Structural barriers presented additional obstacles with the denial of admittance of both partners to treatment programs which had a sole focus on the individual and avoided treating couples.

**Conclusion:**

Detoxification and treatment facilities need to recognize the complex interplay between interpersonal dynamics which shape the treatment experience of couples, and which are also shaped by larger structural dynamics, including barriers in the treatment system. Improvements to the treatment system in general will go a long way in improving treatment for couples. Couples-specific programming also needs to be developed.

## Background

This exploratory study used in-depth interviews and ethnographic engagement to better understand the relationship between interpersonal dynamics and the treatment experience of ten relatively stable drug-using couples in Hartford, CT. The idea for the study grew out of an all-too-frequent predicament. As an ethnographer working on HIV prevention research with street drug users in Hartford, Connecticut, I was often frustrated in my attempts to refer drug users to detoxification and treatment facilities. Single drug users could be placed (at least in detox), although waits were common, but couples were generally not admitted into the Greater Hartford treatment system. Several treatment center gatekeepers with whom I attempted to negotiate entry for couples explained that these unions were "dangerous" and "exploitative." As a result, couples could not attend detoxification or treatment programs together, especially if they were already known to program staff.

Negative stereotyping of drug-using couples is commonplace. Intimate relationships among drug-users, when they are recognized at all [1], have generally been regarded as unscrupulous, unstable and otherwise concerned only with the acquisition and use of illicit drugs [2-4]. Couples research in the substance use field has primarily been conducted among alcoholic spouses and their non-alcoholic wives, and relies heavily on concepts of co-dependence, dysfunctionality and enabling [5].

While research on illicit drug-using couples has been scarce, research on gender differences between injecting drug users (IDUs) has proliferated. This research has pointed out the heightened risk that women IDUs face because they are more likely than male IDUs to acquire HIV sexually [6,7] and to have sexual partners who are injecting drug-users. [8-17]. However, much of this research has tended to ignore the heterogeneity which exists in the types and quality of intimate relationships among drug users and the ways in which these relationships are often valued for more than the material benefits they provide (pooled resources, including drugs) by the high proportion of drug users who are coupled. To give some indication of the proportion of partnered versus non-partnered drug users, in a survey of 601 women drug users (heroin, cocaine and crack users; injectors and non-injectors) in NYC, 71% reported being in a primary heterosexual relationship in the previous 6 months [18].

Treatment research tends to study samples of men and women who are already enrolled in the sex-segregated treatment system, hence this literature rarely considers drug-using couples as a unit of analysis. This exploratory study does. Initially, two questions framed this research: How do interpersonal relationships shape engagement in drug treatment?; and, What are the obstacles drug-using couples encounter in their attempts to enroll, stay in, and maintain treatment outcomes? As the study progressed, an additional question was raised: How are interpersonal dynamics in drug-using intimate partnerships shaped by larger structural dynamics, including structural barriers in the treatment system?

In Principles of Drug Addiction Treatment the National Institute of Drug Abuse (NIDA) recognizes that "no single treatment is appropriate for all individuals"[19] hence treatment settings, intervention models, and related services should be matched to the characteristics of special patient populations. Unfortunately, the NIDA Principles do not address the importance of intimate relationships in the lives of many drug users. The treatment field (like the prevention field) tends to place an emphasis on individuals rather than individuals within their actual social contexts. While there are a growing number of studies on the gender differences of substance users seeking treatment. [20-23] and in treatment settings [24,25], little attention has been given to the experience and complex treatment needs of drug-using couples.

Existing research indicates that drug-using intimate partners are likely to play an important role in determining treatment options [26-28]. These studies stressed the negative role that partnerships assume in relation to treatment experience generally [27,29,30] and the critical role they play in accessing treatment [31,32,4] or maintaining treatment outcomes [33], especially for women.

The nascent field of couple-focused treatment research (where couples are studied as the unit of analysis) has begun to shed light on interpersonal power dynamics between men and women in heterosexual couples and the ways in which these dynamics influence treatment engagement. Riehman et al [34], and other researchers [35], have found that treatment motivation for women drug users may be more influenced by their intimate partners than their male counterparts. In addition, having a partner who had been in treatment increased women's desire for treatment, while having a partner who used drugs but who was not in treatment decreased treatment readiness for women. For both drug-using men and women in intimate partnerships, some degree of economic dependence was also associated with increased motivation for treatment.

McCollum et al [35] examined the association of relationship quality to partner's drug use on treatment outcomes for women. In their sample of 62 couples, the women's perception of relationship quality and their partners' perceptions predicted treatment outcomes. Interestingly, the women reported more days using drugs in post-tests when their partners reported higher relationship quality. Their partners' perceptions of relationship quality were also associated with treatment completion. A higher proportion of women whose partners' reported higher relationship quality (closer, more involved, more satisfying) failed to complete treatment. Finally, women who perceived their relationships to be of lower quality but whose partners perceived them to be of higher quality, were the least likely to complete treatment. In both these studies, the particular ways in which interpersonal dynamics among couples influenced treatment entry, retention and outcomes were left largely unexplained. This exploratory study aims to shed more light on these dynamics, as well as the larger structural forces, including treatment barriers, which appear to play a role in shaping these dynamics.

Rhodes and Quirk [36] identified intimate partnerships as key sites for risk management in individual's drug use and everyday lifestyles. Treatment options are utilized by drug-users as a form of risk management to reduce drug-related and other harms (like incarceration). Understanding how drug users manage risk requires an understanding of the "risk environment" [37]. Drawing on two decades of research on HIV and other health risks among injection drug users. [38-41], Rhodes et al [42] elaborate on this concept. The "risk environment" is the social or physical space in which a variety of risk factors interact to increase the chances of drug-related (or other) harm. The risk environment comprises risk factors exogenous to individuals that serve as direct or indirect barriers to, as well as facilitators of, an individual's HIV and other health risk and preventive behaviors. These environmental factors have been posited as operating at three levels: the micro-level of interpersonal relationships among IDUs; the meso or intermediate level of institutional and organizational responses; and the macro-level of structural forces such as laws, policies, social inequalities, wider cultural beliefs, etc. [43-45]. Furthermore, the risk environment is a product of "interplay" between the three levels: micro, meso and macro which are, in reality, inseparable, and which give rise to "numerous permutations in how environmental factors interconnect in a given context" [42]. This paper utilizes this approach as a heuristic device to further understand the interplay between interpersonal dynamics among drug-using couples, systems of drug treatment, and, broader macro-level forces which constrain the ability of couples to seek alternatives to moderate to high levels of drug use. These three levels – micro, meso and macro – coincide with the three research questions mentioned above.

## Methods

### Study site

The research for this study was carried out at the Hispanic Health Council, Inc., a community-based research and advocacy organization with a long history of involvement in research and intervention projects with illicit drug users in Hartford, Connecticut. Although Hartford is the capital of the wealthiest state per capita in the country, it is the poorest city in the state and one of the nation's poorest per capita for moderate size cities. It has a population of approximately 125,000. According to the 2000 Census, whites comprise 18% of the population, African Americans (including many Caribbeans of African descent) 38%, and Hispanics 40%. Puerto Ricans comprise 89% of the Hispanic population, making it the most Puerto Rican city per capita in the United States [46]. The segregated neighborhoods where Hartford's residents reside are similar to those described in NYC by Wallace as "environments of risk" where urban poverty and political ill will combine to create a "synergy of plagues" [47,48]. A parallel argument was proposed by Singer and Clair [49].

During the study period, drug injection accounted for one half of all new HIV infections and was the most frequent source of new HIV infections nationwide (1.5 infections per 100 injecting drug users per year). Most of these new infections were occurring in cities in the Northeast [50] where AIDS had been the leading cause of death between men and women aged 24–45 years of age. In Connecticut in general, and Hartford specifically, drug use was related to an even larger percentage of AIDS cases than in the nation as a whole. Intravenous drug users, their heterosexual "sex" partners, and their children constituted 61% of the total AIDS cases in the State, as compared to 35% nationally, and 44% in the Northeast. Hartford had annual AIDS rates of 37.1 per 100,000, placing it among the 50 U.S. cities with the highest annual AIDS rates [51]. A new study has estimated that nearly 13% of Hartford's IDUs were HIV positive during this period. This rate of infection ranked Hartford 14^th ^among the largest metropolitan areas in the U.S. [52]. An authoritative estimate of the numbers of IDU's in the Hartford area places this hidden population at 9,600 [53]. Unpublished data indicated that 17.6% of Hartford-area IDUs were enrolled in a program of public or private treatment for injection drug use in 1998. This relatively high level of service ranked Hartford 13^th ^among the 96 largest metropolitan areas in the country (personal communication, Freidman, 2005). Nevertheless, Friedman concluded, "Few if any metropolitan areas seem to be serving IDU populations well" [53].

### Study sample

A sample of 10 drug-using couples were recruited through street outreach and existing projects at the HHC. Two criteria for recruitment were followed for 8 of the 10 couples: At least one member of the couple had to be an injection drug user and both had to use heroin, cocaine, or both, on a daily basis. In two cases, couples were admitted after they were enrolled in methadone maintenance programs. These couples used heroin on occasion but were not daily users. In addition, each member of all 10 couples had to define themselves as a couple and have been sexually involved for at least 6 months. Comparative data which characterizes these 10 couples appear in Table 1.

**Table 1:** Couples Demographics All names are pseudonyms. H = heroin; C = cocaine; CR = crack; A = alcohol. Apt, homeless, Apt means during the course of the study, couples moved from an apartment to being homeless, to an apartment again.

	RACE/ETHNICITY	GENDER	LENGTH OF RELATIONSHIP	RESIDENCE	DRUG USE	HIV/HEP STATUS
CO1: DIANA	B	F	6	APT, HOMELESS	H, C, CR, A	HIV+, HEP C
CO1: GLENN	B	M	6	APT, HOMELESS	H, C, CR, A	HIV+
CO2: SANDRA	PR	F	5	APT	H	HIV-
CO2: JULIO	PR	M	5	APT	H	HIV- HEP C
CO3: DAISY	PR	F	11	ALT W/FAMILY, HOMELESS	H, A	HIV+
CO3: JUAN	PR	M	11	ALT W/FAMILY, HOMELESS	H	HIV+
CO4: PATRICIA	PR	F	4	APT	H,	HIV+
CO4: ANDRES	PR	M	4	APT	H,	HIV+
CO5: LILIA	PR	F	7	APT, HOMELESS, APT	H	HIV-
C05: REINALDO	PR	M	7	APT, HOMELESS, APT	H	HIV-
CO6: RAQUEL	PR	F	22	APT W/2 CHILDREN	H	HIV-
CO6: VICENTE	PR	M	22	APT W/2 CHILDREN	H (SNIFFS)	HIV-
CO7: CANDY	W	F	4	HOMELESS, APT	H	HIV-
CO7: LEONARDO	PR	M	4	HOMELESS, APT	C,	HIV-
CO8: OLIVIA	PR	F	2	HOMELESS	H	HIV-
CO8: SANTO	PR	M	2	HOMELESS	H	HIV-
CO9: ALTHEA	B	M	3	APT	H, A,	HIV+ HEP C
CO9: GEORGE	B	F	3	APT	H	HIV+
C10: DOUGLAS	B	M	7	APT, HOMELESS, APT	H, C, CR, A	HIV+
C10: CHRISTOPHER	B	M	7	APT, HOMELESS, APT	H, C, CR, A	HIV+

As depicted in the table, 12 partners in the study injected heroin only or injected both heroin and cocaine (7 partners). The sole non-injector was a woman who sniffed heroin. The duration of their partnerships ranged from 2 to 22 years (mean = 7.1) at the beginning of the study period. Most of the couples had been together from 3 to 7 years. Three of the couples were African-American, six were Puerto Rican, and one couple was Puerto Rican and white. Their ages ranged from 30 to 51 (most were in their 30's or 40's). Nine of the ten couples were partnered in heterosexual unions. None were legally married. One couple paired two men. Half of the individuals reported being HIV and HCV positive. All couples shared the same serostatus for HIV (1/2 were HIV+, half were HIV-). Three individuals, in three separate couples, reported HCV positivity, but most did not know their serostatus for HCV.

### Procedures

Approval was sought and received from Institutional Review Boards at the Hispanic Health Council, Inc. and Yale University. In addition, a certificate of confidentiality was granted by the federal government. Semi-structured, two-on-one, couple interviews were conducted with each dyad after reading and signing consent forms which clearly described the study as a "couples study." Interviews were conducted in the year 2000. These first interviews explored partnership history, interaction patterns, drug-use behaviors, and AIDS risk behavior. Later, one-on-one, in-depth, open-ended interviews were conducted with each partner about the nature of the relationship; levels of conflict and support within the relationship; survival and "hustling" strategies employed by partners; history of and current drug use; and HIV risk behaviors with their primary partners and others. Drug treatment attitudes and enrollment efforts were also a main focus in these interviews. Whenever possible, the day-to-day realities and contexts of risk were explored by conducting observations of couples in the community. The author also assisted study participants, at their request, to secure beds in detoxification and treatment programs. Fieldnotes were written after each interview, observation or intervention. Study participants were compensated in the amount of $15 per interview. Participants were also compensated for observations if they were set up in advance or involved a substantial time commitment. Participants were not compensated for time spent assisting them to obtain services.

A grounded theory approach [54] was used to inductively code and analyze the nearly 40 transcripts of 60–90 minute interviews and fieldnote data with the aid of Atlas qualitative coding software [55]. Data were analyzed for patterns and associations between characteristics of intimate dyads and their impact on drug use, drug treatment, HIV risk, and other health risks. While the study itself lasted a year, the data accumulated about these couples was actually gathered over a longer period. Some individuals participated in other HHC studies involving the author or otherwise sought her out for referrals and support in times of crisis throughout a six-year tenure at the HHC.

## Results

It is very difficult for impoverished illicit drug users, whether coupled or not, to seriously contemplate treatment options given the constant lure of drugs, the immediate need to alleviate withdrawal symptoms, and the energy and time required for basic survival after drugs have been secured and withdrawal averted. For residential treatment, the logistics are especially complex. Will housing arrangements or a job still be in place once the treatment program has been completed? Where can belongings be stored during the treatment period? For the homeless, what is the use of entering a program if they will simply be put back on the streets without skills or a job once treatment is completed? The need to pay any back bills and "prove" at least a two-year history of heroin use also hinders access to out-patient methadone maintenance programs.

For polydrug users, the additional problem of having to abstain from cocaine use for at least one month and the recognition that they would be terminated from methadone maintenance programs once they failed three urine tests, presented themselves as additional obstacles. Many heroin users knew from past experience that methadone maintenance seemed to increase their desire to use other drugs, primarily cocaine and alcohol (See also Bourgeois [56]).

In addition, for those who have had less-than-positive experiences with treatment, why set oneself up for failure once again? Rather than recognize treatment failure as systemic, or as part of the natural course of recovery, long-term drug users blame themselves. "Once an addict, always an addict." These obstacles and problems are common for impoverished illicit drug users in Hartford who depend on state-funded treatment programs to help manage their addictions. The drug-using couples in this study faced all these challenges and more.

### How did interpersonal dynamics among couples shape engagement in drug treatment?

A companion paper (Simmons and Singer, under review), detailed the ways in which these 10 couples cared for each other. While the relationships between three of the couples were conflictive, and two of these were characterized at times by extreme violence, most intimate partners expressed deeply held feelings for the other, were emotionally invested in and committed to each other, and clearly derived benefits from these relationships. Much of the time, they felt loved and cared for, they felt understood and they valued the companionship of their partners. We detailed how caring for each other in these ways, while managing moderate to high levels of drug use, was not a trivial matter. The lives of these couples were characterized by persistent poverty, the pain of addiction and withdrawal, intermittent homelessness, grief over family members and friends lost to illness, overdose and homicide, their own chronic illnesses (including HIV/AIDS, HCV, depression and anxiety), forced separations due to incarceration, and the stigma attached to addiction, AIDS, and prostitution.

In addition, these 10 couples also cared for each other by helping the other avoid the symptoms of withdrawal. One partner is "sick," the other provides the "cure." Glenn explained why he felt compelled to acquire heroin for Diana.

*G: I care so, I care so much, you know. And our relationship- as far as drugs go- I'd go to any extreme to help her, to keep her from getting sick. She'd do the same for me; at least I've got that feeling*.

His partner, Diana, concurred. She elaborated on this issue when asked what a typical day was like for her:

*D: Waking up needing a bag of dope. Go to the church for breakfast, that's after I have it. If I don't have it, it ain't a typical day 'cause there ain't no getting up until I get it*.

J: So if you can't get up what happens?

*D: Stay there, praying*.

J: Does Glenn go get it?

*D: Yeah. ... He just don't wanna see me like that. He don't wanna see me sick. If you let a person there suffer like that, I don't think. I don't know. I don't know*.

This dynamic of caring for each other and colluding to acquire and use drugs bonded couples together in what were often (but not always) mutually reinforcing cycles of addiction which kept couples from engaging with the treatment system. For many couples, just the idea that one may be in a program while the other was using drugs was out of the question. Couples realized if they were to have even a slim chance of staying clean after detox or treatment, both partners must be clean. Julio and Sandra, who had been together 3 years, described this situation together.

*J: She needs to get herself in a program and I have to get in one too. It is of no use that one is in and that the other isn't. Of what use is it that she is in the program, in treatment, and not me? Then I'm just going to bring her back to the same thing*.

*S: Yeah, 'cause I'll leave the program and go home just to see him get high and then I'm going to want to do it too*.

*J: We'd both just go back to the same thing. Anyone that tells me something different is lying*.

Caring and supporting one's partner also posed problems for couples when one or both were considering entry into a detox or treatment program. Concerns over the stability of the relationship, including fears over the possible loss of the relationship, or the safety of one's partner, were raised as key concerns by couples in this study. Sandra spoke about these issues when the topic of long-term treatment emerged in an interview to help her deal with both her addiction and the recent deaths of 3 siblings to AIDS and hepatitis. *But I worry for him, because I think, what would happen if we ever break up? What will become of him? You know, will he find somebody like me? Somebody that can take care of him? Somebody that would care for him like I do. ... I don't think so*.

In addition, the idea of a partner experiencing dope sickness, or placing oneself in harm's way in order to hustle for drugs, acquire drugs or use drugs due to risks of arrest or overdose made any commitment to enter a program very difficult. Glenn described his views on leaving Diana for a treatment program.

*G: I'm not gonna leave her. I'm not gonna get up and jump in the program right now. We feeling good, and I'm not gonna get into a program with she still out there. That's my bud, you know. I can't let that happen to us. It's just the way I feel. That's the way it is. ... I'm waiting when we both are ready to make a decision to go into treatment 'cause for me to be in treatment and she be out there would worry the hell out of me, you know what I mean? ... She might end up dead*.

On another occasion, Diana waited for Glenn who had to pay an outstanding bill to the program before he could re-enroll.

*D: I got plans, you know and my plans is going into action, but I just want to go in with him. I don't want to be on methadone and he still gotta run out there and go get dope, you know. We gonna try to do this thing together. This is how we always been*.

George delayed his entry into a detoxification program for alcohol because of concerns he had over Althea's emotional state. Althea had recently become very upset after hearing from a friend that people who were uninsured and died from AIDS were buried in body bags in paupers' graves (both George and Althea have AIDS).

*G: Well, right now, I haven't told her, you know what I'm saying? I wanted to find out everything first. Then sit down and talk to her about it. She is at a point right now where she is a little, you know, emotional, especially after hearing about that, you know what I'm saying? So I wouldn't want to break her down. "Oh, everybody is deserting me," or something like that*.

For these couples, caring for each other, and colluding with each other to acquire and use drugs, often took precedence over their concerns about escalating drug use, failing health, and the complex of financial and other worries they contended with on a daily basis. This dynamic of care and collusion also kept them from attempting to access detoxification and treatment services.

### What were the obstacles couples encountered when both sought to engage with drug treatment?

When couples were able to overcome the interpersonal dynamics which kept them from accessing services, and then tried to access them, they encountered the same problems other drug users encountered – a slow bureaucracy, inadequate case management, the inability to deal with the reality of poly-drug use, and the near absence of programs needed to help them deal with persistent poverty, as well as co-occurring mental health issues.

### Couples were prohibited – as couples – from entering the treatment system

In addition, couples who attempted to enter the detoxification and treatment system together were not treated as couples. In a fieldnote written after coordinating referrals to detox for one couple, I noted some of the obstacles we encountered.

At 9:30, Juan and Daisy arrived and told me, "We're ready." They asked me to find them a place where they could detox with methadone. I found out about a facility across the state and located an outreach worker who would take them there. Then I called. They wouldn't take both. I even tried to get it past them by saying I had one man and one woman who were interested in entering their program. They asked, "Are they a couple?" I had to say, "Yes, they've been together for 11 years." The admissions person said, "We can't take both. It's counter-therapeutic," or something like that. Daisy said to Juan, "O.K., you go." Late in the afternoon, I finally found a place for Daisy on the other end of the state (but where they didn't detox with methadone) and found another outreach worker to take her there. Afterwards, Daisy chided Juan, "You got the best place."

Couples were actively prohibited from entering together because their relationships were assumed to be "dangerous" or "exploitative," two other terms I heard when trying to place couples. Nor would treatment programs assist in coordinating services for both partners so that a using partner would not compromise the other partner's attempts to refrain from or limit their drug use upon leaving treatment. While program staff recognized that partners influenced each other, their focus remained on the individual client or patient. Couples understood this prohibition against couples very clearly. Some simply stayed out of the treatment system entirely, like Sandra and Julio. Others tried to coordinate their attempts at treatment with their partners. Without the support of the system, they recognized two additional options: enter under false pretenses ("we're just friends"), or simultaneously enter separate programs when beds were available.

Glenn and Diana, like most of the couples, had utilized all three options: they had stayed out of the system, they had entered under false pretenses, and they had entered separate programs. Glenn felt very strongly about this pervasive prohibition against couples in the treatment field.

*G: I don't agree with it. We are out here together. ... I always felt it was wrong. They think it's unhealthy because of what statistics are saying about two people that were using can't get clean and stay together and be clean. That's how they look at it. Even NA (Narcotics Anonymous) rules, they say, "Don't get into anything serious for a year," or something like that. I don't dig that. Not with someone I've been out there with for five, six years. And then we both decide to get clean, and you gonna tell us not to be together. Fuck you! I don't wanna hear that. That's making a big decision that can wreck a major part of my life, or cause me to go back, you know*.

### When partners entered the treatment system individually, they were not recognized as being part of a couple

While many couples contemplated entering treatment, it was usually a crisis that actually brought them into the system. For example, Glenn and Diana cycled at moderate to high levels of drug use throughout most of the years I knew them. Both had AIDS. Glenn was finally driven into treatment after his infected leg turned black. He went to the emergency room of a nearby hospital. After receiving care for his leg, his doctor told him he might not live if he didn't take better care of himself.

*G: The doctor said my chart don't look good. "I don't see you making it through the end of the year, honestly." She said, "the way you're going, you better think about a program, or think about something other than what you're doing." I ain't wanna tell Diana, because I ain't wanna get her all worked up ...but ... I coulda died*.

The threat to Glenn's health that brought him to the emergency room also catapulted Diana's entry into the treatment system. After leaving the hospital, both enrolled in separate, 21-day detoxification programs. This was a common pattern. When one member of a couple entered the system, propelled by a health crisis like Glenn's or a legal threat like an upcoming probation hearing, the other partner often attempted to enter at the same time. Nevertheless, when partners entered individually, whatever the circumstances, they were not recognized as part of a couple. Yet couples' dynamics still played out in the hearts and minds of partnered drug users. Consider, for example, what happened to Glenn. Both he and Diana entered treatment at the same time, but the story didn't end there. Both also left early. In a joint interview, Glenn explained his truncated stay.

J: So why didn't you stay longer?

*G: I would of 'cause it kept lingering in the back of my mind what the doctors was telling me. But I was concerned because I didn't know what was happening with my other half out there. I didn't have no outside communication with Diana, to know what was going on. That woulda been a help*.

Although Diana had followed Glenn into another detoxification program, he didn't know it at the time. Neither knew where the other had been placed. It turned out that Diana only stayed for 4 days (for reasons explained below), but the lack of communication between the two of them weakened Glenn's determination to complete the 21-day program. His concerns for Diana's safety played a significant role in his decision to leave the facility (as well as, perhaps, his assumption that she was using and his desire to use with her.)

A similar dynamic characterized Daisy's treatment experience. Daisy and Juan had been together 11 years. He avoided treatment, but jail provided him regular intervals of respite from moderate to high drug use. During the years I knew this couple, Daisy often tried to enter treatment once Juan was jailed. Ultimately, however, when he was released, both would relapse and resume their pattern. One of the last times that Juan was incarcerated, Daisy enrolled in a long-term treatment program. She did well for 9 months and had high expectations of regaining custody of their children. Her effort failed, however, when she left on a pass to visit Juan only days after his release. She missed the bus that would have brought her back to the program on time. Juan described what happened next.

*J: Daisy called and they told her that she would lose points or whatever [for being late]. They would drop her down, and she got mad. She said to them, "You knew that I had to be back to this program on Sunday. How come you didn't have a ride already for me on Sunday?" Then she said to me, "I'm staying with you." She said she wasn't gonna go back to drop her points. She was at a level where she had gotten so far in that program. Hogar Crea [the residential program] gave her $150 for shopping clothes, so when I took her to Bradlees to shop, she bought me some pants and bought her some cosmetics and all that. An hour later we were selling the clothes [so we could buy dope]*.

While treatment programs have their own rationale for rules and regulations, the unwillingness of these programs to fully recognize the powerful interpersonal dynamics that shape patterns of drug use and patterns of treatment engagement, leaves couples to their own devices and survival strategies. The question begs to be asked: How might treatment programs take advantage of the precipitating crises which often bring couples or individual partners into the treatment system, as well as act on the recognition that, ultimately, most partners will reunite. Simply discouraging drug users from resuming relationships with other drug users, including intimate partners, upon the termination of treatment, underestimates the importance these relationships have for drug users.

### How were interpersonal dynamics in these drug-using intimate partnerships shaped by larger structural forces, including structural barriers in the treatment system?

The couples participating in this study demonstrated how interpersonal dynamics, such as care and collusion, shape drug use and treatment experience. These interpersonal dynamics, however, were not patterned randomly, but took shape in the shadow of larger structural forces. Gender relations, including gender inequality, poverty and the never ending "War on Drugs" all factored into the lives of these couples: the ways in which they fell into their addictions, their HIV status (for half of the participants), and their continuing struggle to survive amidst the discrimination and other stigmas they experienced as poor Blacks and Puerto Ricans, drug users and felons. More than half of the 20 individuals who participated in this study had spent time in jail. In nearly every couple, at least one partner had lost custody of their children to the state or to family members. Their stake in conventional society was severely circumscribed. The treatment system had few resources and proved to be inadequate at addressing these larger structural forces, and at times, appeared even to reproduce them.

### Persistent poverty shaped interpersonal dynamics and the ability to take advantage of treatment options

Poverty has been a constant in the lives of all ten couples. Several couples in this study met as teenagers and started using drugs around the same time. For most, siblings, cousins, peers, and especially for women, boyfriends, turned them on to cocaine, crack and/or heroin or otherwise piqued their curiosity. Drugs and drug selling were ubiquitous and illegal means of employment (drug dealing, theft, and sexual exchange) were much more lucrative than the kinds of low-paying, low-status jobs that were available.

Juan and Daisy were one of the couples who met as teenagers and have been together ever since. Juan recognized how their addictions were propelled by his drug dealing and a constant access to drugs. Yet, he also felt that his educational deficits, the stigma of addiction, and the legal consequences of his status as a felon, limited his ability to compete for the low-paying, low-status jobs available to him – jobs which he felt were demeaning.

*J: I go out there and I'm selling the drugs. I'm coming back home with money and the drugs is on me. She's gonna see and she wants it. She knows she's sick. She can't do nothing but do this needle just to get normal. And I'm the one who provides the needles for her. I wanna go back to school but right now I am not gonna get caught in no McDonalds and I'm not gonna get caught in no Walgreens or nothing like that. Jobs that I want is not out there or not available for me yet. I need education maybe, but I wanna be a security guard. I can't do that until about four or five years. Or at least I wanna be going to some kind of job with a suit and tie, or a tie and some baggy pants. I don't wanna be going to no McDonalds, it's just not me. If it's not out there what I want then, I'm just gonna have to take the risk in doing what I do*.

Juan recognized that the interpersonal dynamics which propelled his and Daisy's addictions were shaped by larger structural forces – a lucrative drug market in Hartford, the lack of jobs which paid a sustainable wage, and the stigma of being an ex-felon and an addict.

Julio made many of the same choices Juan had made. Like Juan, he had spent many years in jail as a result. But Julio was adamant about avoiding a return to jail and limited himself to only legal hustles – like picking up cans with Sandra – to supplement his monthly Workman's Compensation check. When Julio wanted to turn his life around upon release from jail, he ventured to Hartford compliments of the same migrant agricultural program which brought the first wave of Hartford's majority Puerto Rican population to the mainland – to work in the Connecticut Valley's tobacco fields. His dream of establishing himself as a legitimate wage-earner disappeared when he fell ill with diabetes and hepatitis C – diseases which made work in the fields impossible. Still, Julio's Workman's Compensation check, like the SSI payments or Veteran's Benefits several other men in the study received, afforded him an income which he could count on to pay the rent for a room or small apartment. Sandra was able to supplement his meager income with food stamps.

The six couples who were entitled to at least one of these programs were homeless less often. The one exception was a couple where both partners suffered from co-occurring mental health disorders. One of these partners was diagnosed with schizophrenia. The six couples who received some benefits from entitlement programs also tended to enroll in treatment programs more often. But these entitlements were not always enough to keep couples from the brink of disaster. Continuing the story of Glenn and Diana's truncated stay in the treatment program, Diana left her detoxification program early due to both a shortage of beds in the 21-day detox, as well as concerns over threatened homelessness and the loss of all of the couple's personal belongings. A previous arrest (both she and Glenn were arrested and did time) had already traumatized them to this eventuality. They described the circumstances around her decision to leave treatment.

*G: Diana only stayed 4 days. That was just a detox for her 'cause they didn't have any beds upstairs (in the 21 day program)*.

*D: Plus, I was scared 'cause I got this letter saying they was gonna put our stuff out on the street. And it wasn't putting our stuff out for non-payment of rent, either*.

*G: That letter came and that letter scared Diana*.

*D: Now I'm thinking all about this while I'm in the program. Let me show this to Janie*.

J: [author reading] "We are renovating the Avon hotel as a result we will be closing some of the rooms and requiring tenants to relocate. All rental agreements are weekly, therefore we will not renew the rental agreements for the rooms under renovation. By law, we are allowing you a week to relocate and remove your belongings from the premises. You are required to pay one half of your rent, one hundred and seventy, and keep the other half to allow you the finances to relocate to another facility. Your rental agreement will not be renewed as of Wednesday, May third, and you will be required to remove your belongings on May tenth. Your room will be locked as of May tenth. We will not be responsible for any property." So what happened?

*D: I'm not dumb. I know you're supposed to get some kind of stupid papers served to you*.

J: So you went to legal aid?

*D: Yeah I did, because the way we're living it's a health hazard anyways for both of us. There's nothing but dust and dirt in the room. I mean we're never eating right. I think it's a godsend for us to have to leave outta there*.

*G: I only want to stay there, because we have to. Not because we want to, you know*.

D: I don't want to be there anyways!

*G: I only want to be there because we really, honestly, have nowhere else to go*.

Glen's entitlement to VA benefits had secured them sub-standard housing which was preferable to homelessness. The impending loss of their housing due to gentrification compromised Diana's ability to take advantage of the treatment that was available to her. By the time Glenn arrived home, she had already relapsed and had become embroiled in a legal dispute with the landlord. He, of course, relapsed as well and was forced to spend his time searching for alternative housing.

### Aversion to particular treatment modalities played a role in shaping interpersonal dynamics by limiting treatment options

The incarceration of men, in particular, often brought their partners into the treatment system, but the system was not especially adept at keeping them there. One other way in which the punitive nature of incarceration influences the choices couples make concerning treatment involves methadone and the way in which methadone is dealt with in the criminal justice system in Hartford, as well as the clinics themselves. Several couples opted out of methadone maintenance, or avoided it as much as possible, because they feared the consequences of being arrested. None of the couples, even when they enrolled in methadone maintenance programs, abstained entirely from using heroin or other illicit drugs, most notably cocaine or crack. Arrest was therefore always a possibility. And arrest would ultimately mean "kicking" methadone "cold-turkey" in jail. Withdrawal from methadone was always described as an extremely painful and disorienting experience. Andrés related this horror.

*A: Methadone is real hard to kick. I almost dies trying to kick that*.

J: In jail?

*A: Yeah I almost flipped you know, no not died, but almost flipped*.

J: Mentally?

*A: Yeah. It is real depressing. It's like, I don't know, it is something inside of you to be shaking like that. Oh man, that's worse than kicking dope. You can't sleep for like 17 days or so. You be up, everybody be sleeping, and it's like 17 days straight you can't sleep. You try to go to sleep and you can't. And you be jerking, Oh man. That's why I'm scared to go back in it. I never experienced something like that*.

Aversion to a particular treatment modality was not confined to methadone maintenance. Julio related his opposition to the philosophies of the two main long-term residential treatment programs available to him and Sandra in the Greater Hartford area. This aversion was also largely born of experience.

*J: I'd rather go to jail than go to Hogar Crea or Teen Challenge. Hogar Crea is like an army. I was there many months [in Puerto Rico] and they treat you badly. They discipline everyone if just one person does something wrong. They give you the same punishment. ... I understand that I am an addict like all the others. But I didn't go there to put up with this kind of craziness from someone who is an addict just like me. Or to be shouted at, or spit at in the face. This is humiliating. I have an addiction, I don't deny that. But that they treat me like that? No. Teen Challenge is religious. From when you get up in the morning to when you go to bed at night, they have you reading the Bible . without any medication, just vitamins. They wrap you up in the Bible, in God's word. You come out of there a priest! But once you go out into the street, it's back again to the same thing*.

Julio's aversion to the two residential treatment programs mentioned above also impacted Sandra's choices. She was not inclined to enter a treatment program unless he went also. In addition, his prior experience with different treatment modalities influenced her thinking about what modality would be most appropriate. The lack of a larger range of treatment modalities available to Hartford addicts, together with their lack of understanding of where they could be placed left Julio and Sandra to choose between what they perceived as a highly religious approach to treatment or an extremely humiliating one. They opted for none of the above.

Detoxification was the option most couples utilized to help them manage their addictions. This reliance on detoxification programs rather than more comprehensive harm reduction or treatment programs dramatically increased the chances that couples would resume their former drug use and feel like failures once again. This sense of failure also translated into increased drug use. Many couples equated detoxification with treatment, and failure was seen as a failure of will, not as a failure of the system, or as part of the normal course of recovery. Andrés described his addiction and blamed himself. He also described the dangerous position his addiction places him in.

*A: It's me, you know. I know I'm the problem. I could be feeling real good, nice and sober but then I want to get high. I won't think about it twice. I can't stop. I wanna stop but I can't. I wish I could just say, I'm not going to shoot no more. That's what I would like to do. But, I can't do it. I could go for a little while, but after the little while, I wanna go do some dope. It's like right now I am sick, and I haven't gotten that sick, but in like an hour from now I am going to get sicker and sicker and I am gonna want some dope, and I am gonna do anything to get that dope. It's always the same, and the same and the same. And now I just beat these guys for $350. Oh God, I gotta watch myself*.

Julio, who by comparison, is doing better than most of the couples in this study, despite his reluctance to engage with treatment programs, recognizes how difficult it is to free oneself from addiction in the midst of persistent poverty. He relates how he took Sandra to detox when they first met and how difficult leaving detox was when you do not return to a home but to a homeless shelter.

*J: If there was a program, I'd like to get into it because this life isn't any good. I'm tired. I believe that if I could get clean, I wouldn't turn back. I don't know; I can't really say. I really wouldn't dare say because there's times when one makes promises and they can't be kept. I wouldn't like to return to it, but ... I realize that there are so many people who are in these programs and they go to meetings and then when they get out, they just go drink and drug themselves. It's really difficult. The thing is ... I took Sandra to ADRC when she and I started being together. Sandra drank also so I took her there. She was there for 5 days and then I went and got her. She came out alright. She was clean for quite a while. It was nice. But we were living in the shelters and that makes it really hard. One really never gets accustomed to that. It was really difficult for Sandra*.

## Discussion

This exploratory study emphasized the importance that intimate partnerships have for the 20 men and women who participated in this study. Even though several of these relationships were conflictive (two were actually characterized by extreme violence), all of these relationships involved complex interpersonal dynamics which shaped drug and sexual risk, as well as treatment engagement. Most of these relationships, however, were characterized by "care" and a commitment to preserving the relationship, despite the many hardships, and in many ways because of the hardships, these couples endured. They were also characterized by collusion, whereby intimate partners colluded with each other to acquire and use drugs in what was often, but not always, a mutually reinforcing cycle of addiction. This dynamic of care and collusion may help to explain findings such as those presented by McCollum et al [35] where perceptions of relationship quality predicted treatment outcomes.

The interpersonal dynamic of care and collusion operated at the micro level of environmental risk and shaped treatment engagement, as well as drug-related and other health risks, by keeping drug-using couples out of the detoxification and treatment system. But this dynamic was also shaped by the risk environment operating at the meso level due to the structural barriers which prohibited couples from entering detox or treatment together, or the inability to recognize the ways in which interpersonal dynamics influenced partners entering the system as individuals.

Almost all couples decried the refusal of treatment systems to coordinate the detoxification and treatment needs of drug-using intimate partners in tandem so that one partner would not be a threat to the other's efforts to stay clean or reduce drug use when s/he returned home.

Other meso level structural barriers influenced treatment engagement for couples. Two-thirds of the couples in this study utilized 3–5, or 21 day detoxification programs, methadone maintenance programs, and residential treatment to reduce their tolerance for drugs, either in the short or the long term. One-third opted out of treatment entirely due to past negative experiences with particular treatment modalities, especially methadone. The forced withdrawal from methadone in jail resulted in conflict and delays in engaging with methadone maintenance programs, even when drug use tolerance escalated to unsustainable levels. The interplay between the criminalization of drug use and the reluctance of methadone maintenance programs to deal with polydrug use is evidenced in this study. This interplay also impacted the partners of drug users who had experienced extended and painful withdrawals from methadone in prison. The conflicts and delays of one partner often resulted in delayed treatment for the other partner because both preferred to enter treatment sites together, whenever possible.

The repeated incarceration of men, in particular, often brought their partners into the treatment system, but the ability of these women to remain in the system, or to continue a pattern of reduced drug use once their partners were released, was limited. One can only wonder if Daisy and Juan would have fared any better if Juan had actually been sent to a treatment program rather than prison, or received treatment services in prison, or been referred to a treatment program, perhaps the same one as Daisy, upon his release. Or, what if the illicit drugs that these couples consumed were regulated rather than illegal, and they could utilize heroin maintenance programs like those operating in some other countries? How much better would Sandra and Julio's life have been then?

Several studies have pointed out the limitations and weaknesses of treatment programming and the severe underfunding that treatment receives relative to other facets of the "War on Drugs". [56-59]. Singer notes that of...

*$18 billion in the federal drug budget, only one-third is directed towards prevention and treatment efforts. However, only 10 percent of the approximately $6 billion of federal money targeted to reduce the demand for drugs is earmarked for the treatment of the estimated 4 million hardcore drug users in the United States *[59].

The interplay between larger social forces, such as the "War on Drugs," which cause funding disparities and criminalizes drug use and drug users ultimately shapes the experience of couples at the micro level of environmental risk. This disparity in the distribution of funds is particularly discouraging when the social benefits of drug treatment (even inadequate treatment) are so well supported in the evidence-based research literature. Out-of-treatment injection drug users are more likely to be infected from HIV than drug users in treatment, and the longer one stays in treatment, the longer one is likely to be able to reduce both drug use and risk from HIV infection

[60,61,59].

## Conclusion

Drug-using couples are first and foremost drug-users, and improvements to the treatment system in general will go a long way to improving treatment for couples. See Shavelson [58] for a list of recommendations that would resonate with many of the concerns voiced by couples in this study. Drug-using couples also bring unique needs to the treatment system. Although treatment programs attempt to avoid the difficulties associated with couples, it is likely that a majority of the individuals seeking treatment are involved in long-term relationships. This is particularly true of women who are more likely to be partnered with drug-using men [13,17,62]. The refusal of treatment programs to deal with couples, therefore, disproportionately impacts women drug users.

In order to assist couples find alternatives to moderate to high drug use, a better understanding of the complex interpersonal dynamics which shape drug-related and other health risks is needed. The following couples-specific recommendations would help support drug-using couples manage their addictions, as well as their health and other needs more successfully.

1) Recognize drug-using intimate partnerships at entry in the treatment system.

2) Recognize the heterogeneity that exists among drug-using intimate partnerships, and the importance that these relationships have for many couples.

3) Recognize the complex interpersonal dynamics among couples that shape treatment entry, retention and the ability to maintain treatment outcomes.

4) Make couples counseling a common and expected add-on in treatment programming.

5) Recognize the ways in which interpersonal dynamics are shaped by larger social forces, including structural barriers and inadequacies in the treatment system.

6) Enable couples to opt for a wider enhanced variety of treatment options, as well as enhanced social support (housing assistance and placement, job skills training and placement, parenting skills training and support, and family reunification services).

7) Negotiate treatment options with couples that are in the best interests of couples on a case-by-case basis. Clearly, couples whose relationships are characterized by intimate partner violence may need to exercise different options than couples whose relationships are not characterized by violence.

8) Finally, recognize that treatment improvements alone cannot reverse the impacts of persistent poverty, racism and gender inequality. The fostering of structural changes at all levels – micro, meso and macro – are needed in order to alleviate the social vulnerabilities which make drug use and drug-related harms so pervasive and so harmful among the poor.

## Competing interests

The author(s) declares that she has no competing interests.

## Authors' contributions

N/A
